# Overweight in patients with chronic obstructive pulmonary disease needs more attention: a cross-sectional study in general practice

**DOI:** 10.1038/s41533-017-0065-3

**Published:** 2017-11-22

**Authors:** Lisa D. M. Verberne, Chantal J. Leemrijse, Ilse C. S. Swinkels, Christel E. van Dijk, Dinny H. de Bakker, Mark M. J. Nielen

**Affiliations:** Netherlands Institute for Health Services Research (NIVEL), Utrecht, The Netherlands

## Abstract

Guidelines for management of chronic obstructive pulmonary disease (COPD) primarily focus on the prevention of weight loss, while overweight and obesity are highly prevalent in patients with milder stages of COPD. This cross-sectional study examines the association of overweight and obesity with the prevalence of comorbid disorders and prescribed medication for obstructive airway disease, in patients with mild to moderate COPD. Data were used from electronic health records of 380 Dutch general practices in 2014. In total, we identified 4938 patients with mild or moderate COPD based on spirometry data, and a recorded body mass index (BMI) of ≥21 kg/m^2^. Outcomes in overweight (BMI ≥ 25 and <30 kg/m^2^) and obese (BMI ≥30 kg/m^2^) patients with COPD were compared to those with a normal weight (BMI ≥ 21 and <25 kg/m^2^), by logistic multilevel analyses. Compared to COPD patients with a normal weight, positive associations were found for diabetes, osteoarthritis, and hypertension, for both overweight (OR: 1.4–1.7) and obese (OR: 2.4–3.8) patients, and for heart failure in obese patients (OR: 2.3). Osteoporosis was less prevalent in overweight (OR: 0.7) and obese (OR: 0.5) patients, and anxiety disorders in obese patients (OR: 0.5). No associations were found for coronary heart disease, stroke, sleep disturbance, depression, and pneumonia. Furthermore, obese patients were in general more often prescribed medication for obstructive airway disease compared to patients with a normal weight. The findings of this study underline the need to increase awareness in general practitioners for excess weight in patients with mild to moderate COPD.

## Chronic lung disease: Watching weight in the early stages

Doctors should pay more attention to weight management in patients with mild chronic lung disease, say researchers in the Netherlands. While guidelines for treating chronic obstructive pulmonary disease (COPD) focus primarily on preventing weight-loss in severe COPD, around 65 per cent of patients with mild COPD are overweight or obese. Excessive weight exacerbates symptoms and triggers co-morbidities, and so can impact heavily on COPD healthcare provision. Lisa Verberne and co-workers at the Netherlands Institute for Health Services Research analyzed data from 315 Dutch general practices in 2014, identifying 3404 patients were classified as overweight or obese and 1534 as normal weight. So in total 4938 patients were identified. The team compared this cohort with COPD patients of normal weight, and found they were at higher risk of diabetes, osteoarthritis and hypertension. Obese patients were also at risk of heart failure and were prescribed more medication for COPD.

## Introduction

Chronic obstructive pulmonary disease (COPD) is a highly prevalent chronic disease.^1^ Although weight loss is common in patients with COPD, previous studies have shown that about 65% of the COPD population is overweight or obese.^2–5^ Obesity is a well-known risk factor for several diseases, such as diabetes mellitus and cardiovascular diseases, also in patients with COPD.^6,7^ Moreover, obesity in patients with COPD is associated with several other health consequences, like increased symptoms of dyspnea, a higher prescription rate for inhaled medications, and increased healthcare utilization.^3,8–10^ Nevertheless, the global initiative for chronic obstructive lung disease (GOLD) that provides evidence for the assessment, diagnoses, and treatment of COPD, primarily focus on the prevention of weight loss,^11^ as underweight in patients with COPD is associated with a higher risk of all-cause mortality.^12^ However, this mostly applies to patients with severe COPD where an increasing body mass index (BMI) is linearly associated with a better survival, while in patients with mild to moderate COPD the lowest mortality risk occurs in normal to overweight patients.^13,14^

Since both COPD and obesity places a high burden on the healthcare system, it is important to gain more knowledge on the clinical profile of overweight and obese patients with COPD. Previous studies that investigated the implications of overweight and obesity on health outcomes were conducted only in the overall COPD population, including patients with severe COPD.^3,4,8–10^ However, in patients with COPD, excess weight is mainly present among those with milder stages of COPD.^15^ These patients are generally treated in primary healthcare, and it therefore seems relevant to study the association of weight and health outcomes specifically in patients with mild to moderate COPD. This knowledge can contribute to the development of appropriate treatment strategies for patients with COPD in primary healthcare.

The aim of the current study is to determine the association of overweight and obesity on the prevalence rate of comorbid disorders and prescribed medication for obstructive airway disease in patients with mild to moderate COPD in general practice.

## Results

**Fig. 1:**
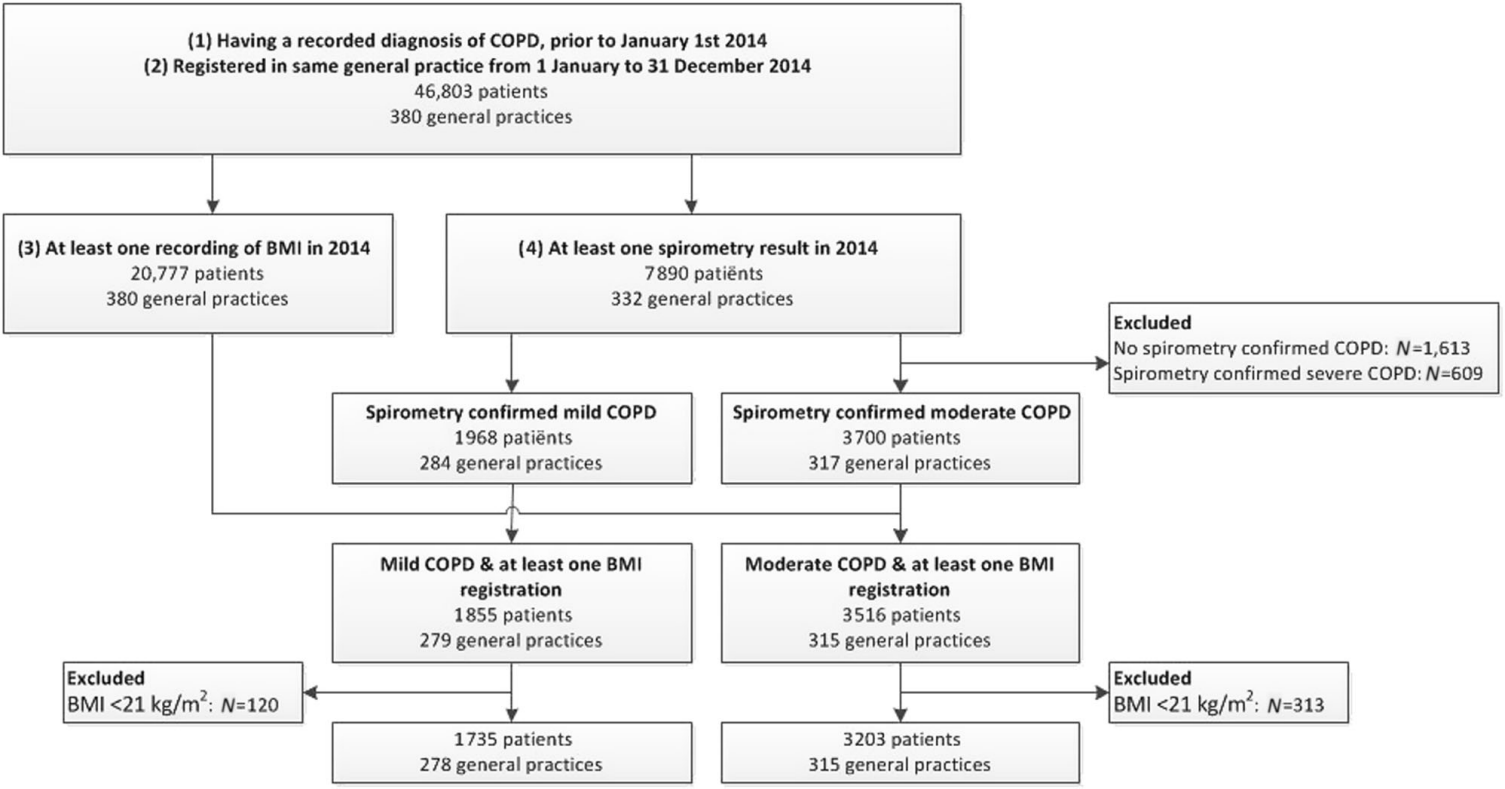
Flow diagram of patient selection. COPD chronic obstructive pulmonary disease, BMI body mass index

Initially 46,803 patients were detected in the NIVEL Primary Care Database (NIVEL-PCD) with a diagnosis of COPD prior to 1st January 2014, of which 20,777 (44%) had a BMI recorded and 7890 (17%) had a spirometry result in 2014. After applying all selection criteria (Fig. 1), 4938 patients with mild to moderate COPD were eligible for inclusion in the current study. The final study population consisted of about one-third of patients with mild COPD and two-third of patients with moderate COPD. Table 1 shows the characteristics of the study population. In total, 54% of the patients were men, mean age was 67 years, and mean BMI was 27.5 kg/m^2^.

**Table 1:** Characteristics of patients with mild to moderate chronic obstructive pulmonary disease

	Normal weight	Over weight	Obesity	Total
Patients (*N*)	1534	2212	1192	4938
Gender, % men	47.3	60.3	51.9	54.2
Age, mean (SD)	66.9 (10.7)	68.1 (10.3)	66.6 (10.2)	67.3 (10.4)
BMI, mean (SD)	23.2 (1.1)	27.2 (1.4)	33.7 (3.7)	27.5 (4.4)
FEV_1_ % predicted, mean (SD)	75.1 (14.8)	75.5 (14.4)	74.0 (14.1)	75.0 (14.5)
*Smoking status (% patients)*				
Never	9.5	8.5	8.8	8.9
Former	39.3	56.1	57.3	51.2
Current	51.2	35.4	33.9	40.0
*Comorbid disorders (% patients)*				
Coronary heart disease	3.9	5.2	4.7	4.7
Stroke	7.0	8.6	7.4	7.8
Hypertension	36.4	44.1	56.2	44.6
Heart failure	3.7	4.6	6.8	4.8
Osteoporosis	11.2	7.7	6.2	8.4
Osteoarthritis	14.8	19.4	26.7	19.7
Sleep disturbance	5.2	5.8	5.2	5.5
Anxiety disorder	3.6	2.5	1.6	2.6
Depression	6.4	5.1	5.5	5.6
Pneumonia	5.2	4.3	4.5	4.6
Lung carcinoma	1.2	0.9	0.4	0.9
Diabetes	11.3	18.0	31.2	19.1
*Medication (% patients ≥1 prescription)*				
SAMA	8.3	7.9	8.9	8.3
SABA	20.9	24.0	28.4	24.1
LAMA	42.2	45.2	48.2	45.0
LABA	11.9	13.1	13.8	12.9
ICS	12.5	13.7	11.4	12.8
LABA + ICS^a^	43.3	42.6	48.9	44.4
Prednisone	20.0	20.0	22.3	20.6
Antibiotics	26.8	25.2	27.1	26.1

*SAMA* short-acting muscarinic antagonist, *SABA* short acting beta2-antagonist, *LAMA* long-acting muscarinic antagonist, *LABA* long-acting beta2-antagonist, *ICS* inhaled corticosteroids

^a^ medication with a combination of LABA and ICS

### Comorbid disorders

**Fig. 2:**
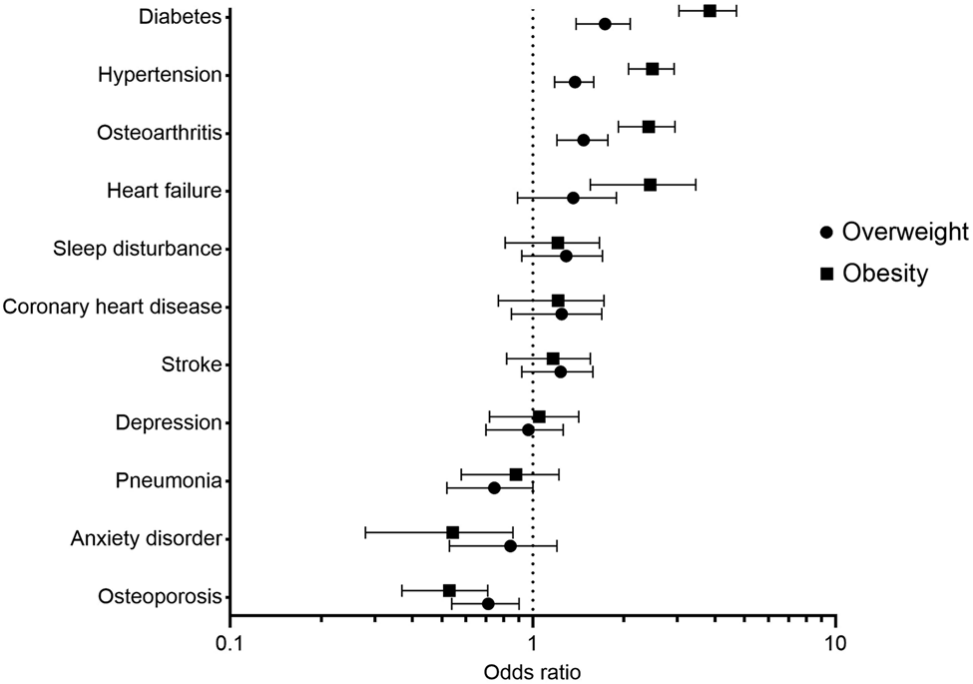
Adjusted odds ratios for the association of weight and comorbid disorders in patients with mild to moderate chronic obstructive pulmonary disease. The black dots represent the odds ratios (ORs) for the prevalence rate of comorbid disorders in overweight and obese patients, using the normal weight patients as reference category. The ORs are adjusted for clustering effect of general practice, gender, age, smoking status, and lung function. The error bars represent the 95% confidence intervals around the ORs

In all weight categories hypertension, osteoarthritis, and diabetes are the highest prevalent comorbid disorders (Table 1). For the comparison of overweight and obese patients with the normal-weight patients, adjusted odds ratios (ORs) for comorbid disorders are shown in Fig. 2 for the main analyses (also see Supplementary Table 1). Only comorbid disorders with a prevalence rate of at least 1% were evaluated. The strongest positive associations were found for obese patients, subsequently for diabetes (OR: 3.79; 95% CI: 3.04, 4.71), hypertension (OR: 2.46, 95% CI: 2.07, 2.93), osteoarthritis (OR: 2.38; 95% CI: 1.92, 2.95), and heart failure (OR: 2.32, 95% CI: 1.55, 3.46). Significant inverse associations were found for osteoporosis (OR: 0.51; 95% CI: 0.37, 0.71) and anxiety disorders (OR: 0.49; 95% CI: 0.28, 0.86). No significant associations were shown for coronary heart disease, stroke, sleep disturbance, depression and pneumonia with weight category. Interaction effects for BMI-category and smoking were shown in the associations with osteoarthritis, anxiety disorders, and depression. For osteoarthritis ORs were higher for both overweight (*p* for interaction = 0.09) and obese patients (*p* for interaction = 0.06) who were never or former smokers, as compared to current smokers. For anxiety disorders the OR was lower for overweight patients who were never or former smokers (*p* for interaction = 0.07), and for depression the OR was lower for obese patients who were never or former smokers (*p* for interaction = 0.03), as compared to current smokers. Interaction effects for BMI-category and COPD-status were shown for obese patients only. For obese patients with mild COPD, the associations were more positive for heart failure (*p* for interaction = 0.08), and more negative for coronary heart disease (*p* for interaction = 0.03) and depression (*p* for interaction = 0.05), as compared to obese patients with moderate COPD.

### Medication

In total, 88% of the patients was prescribed at least one medication for obstructive airway disease in 2014. Almost half of the patients were prescribed long-acting muscarinic antagonist (LAMA) and long-acting beta2-antagonist (LABA) + inhaled corticosteroids (ICS). About a quarter of the patients were prescribed short acting beta2-antagonist (SABA), prednisone and antibiotics. Short-acting muscarinic antagonist (SAMA), LABA, and ICS were less prescribed (Table 1). Table 2 shows the ORs for the main analyses on the association of BMI-category and medication. Both overweight and obese patients were prescribed significantly more often SABA as compared to normal weight patients. Moreover, obese patients were significantly more likely to be prescribed LAMA and LABA + ICS. For the association of BMI-category with SAMA, ORs for obese patients were higher for current smokers than for never or former smokers (*p* for interaction = 0.07). Interaction effects for BMI-category and COPD-status were shown in the associations for SABA, LABA, prednisone, and antibiotics. For these medication-classes, associations for obese patients with mild COPD were more positive as compared to obese patients with moderate COPD. The strongest interaction effect was shown for prednisone (*p* for interaction <0.01), showing a significant association with obesity for patients with mild COPD (OR crude model: 1.7), but not for patients with moderate COPD (OR crude model: 1.0).

**Table 2:** Odds ratios for the association of weight and prescribed medication for obstructive airway disease in patients with mild to moderate chronic obstructive pulmonary disease

		Normal weight *N* = 1534	Overweight *N* = 2212	Obesity *N* = 1192
≥1 prescription SAMA	No. cases	128	175	106
	Model 1	ref.	0.94 (0.74–1.20)	1.07 (0.82–1.40)
	Model 2	ref.	0.96 (0.74–1.24)	1.13 (0.84–1.51)
≥1 prescription SABA	No. cases	321	530	339
	Model 1	ref.	1.19 (1.02–1.39)	1.50 (1.26–1.79)
	Model 2	ref.	1.26 (1.06–1.50)	1.55 (1.28–1.89)
≥1 prescription LAMA	No. cases	647	999	574
	Model 1	ref.	1.13 (0.99–1.29)	1.27 (1.09–1.48)
	Model 2	ref.	1.13 (0.97–1.31)	1.24 (1.05–1.47)
≥1 prescription LABA	No. cases	183	289	164
	Model 1	ref.	1.11 (0.91–1.35)	1.18 (0.94–1.48)
	Model 2	ref.	0.98 (0.79–1.22)	0.99 (0.77–1.28)
≥1 prescription ICS	No. cases	192	302	136
	Model 1	ref.	1.11 (0.91–1.34)	0.90 (0.71–1.14)
	Model 2	ref.	1.03 (0.83–1.27)	0.78 (0.61–1.01)
≥1 prescription combination LABA+ICS	No. cases	664	943	583
	Model 1	ref.	0.97 (0.85–1.11)	1.25 (1.08–1.46)
	Model 2	ref.	1.01 (0.88–1.18)	1.31 (1.11–1.56)
≥1 prescription prednisone	No. cases	307	442	266
	Model 1	ref.	1.00 (0.85–1.17)	1.15 (0.95–1.38)
	Model 2	ref.	1.05 (0.88–1.25)	1.20 (0.98–1.47)
≥1 prescription antibiotics	No. cases	411	557	323
	Model 1	ref.	0.92 (0.79–1.07)	1.02 (0.86–1.20)
	Model 2	ref.	0.93 (0.79–1.09)	1.02 (0.85–1.23)

Odds ratios are presented with their 95% confidence interval

Model 1: crude model (*N* = 4938)

Model 2: adjusted for clustering effect of general practice, gender, age, smoking status, and lung function (*N* = 4583)

*SAMA* short-acting muscarinic antagonist, *SABA* short acting beta2-antagonist, *LAMA* long-acting muscarinic antagonist, *LABA* long-acting beta2-antagonist, *ICS* inhaled corticosteroids

## Discussion

### Main findings

The present study illustrated that overweight, and to a greater extent obesity in patients with mild to moderate COPD, is associated with a higher prevalence rate for the most dominant comorbid disorders, i.e. hypertension, osteoarthritis, diabetes, and with a higher prevalence rate for heart failure in obese patients, as compared to patients with mild to moderate COPD and a normal weight. Osteoporosis and anxiety disorder were inversely associated with overweight and obesity. Furthermore, obesity was associated with increased prescription of medication for obstructive airway disease.

This study examined the association of weight and health outcomes, specifically among patients with mild to moderate COPD. The findings on comorbid disorders complement to results of previous observational studies that were conducted in the general COPD population, regarding diabetes,^2–4,6–9^ hypertension,^2,6–9^ osteoarthritis,^9^ and osteoporosis.^9^ Regarding cardiovascular diseases, we found positive associations for obesity and heart failure, but not for coronary heart disease and stroke. Findings for cardiovascular diseases in previous studies were not conclusive. Lambert et al.^9^ found obesity to be associated with an increased risk of coronary heart disease and heart failure, while Cecere et al.^8^ did not. Other studies demonstrated that a higher BMI was associated with a higher risk of several cardiovascular diseases.^2–4,6^ However, results on cardiovascular diseases were difficult to compare since different definitions for cardiovascular diseases were used, and most studies were based on self-reported data.

Based on previous studies in the general population we would have expected positive associations of weight and mental health problems.^16,17^ However, we found mental health problems to be negatively (anxiety disorders), or not (sleep disturbance and depression), associated with increasing weight. These results also deviate from findings of a previous study conducted in a COPD population, showing a positive relation of obesity and obstructive sleep apnea.^9^

The association of weight and prescribed medication for obstructive airway disease is a less studied subject. Only two previous study evaluated this association in a COPD-population and found that overweight or obese patients were prescribed more often SABA, LABA, and ICS.^3,8^ These results are in agreement with the findings of our study. Moreover, in our study we showed that the association of obesity and prescribed medication was most strongly in patients with mild COPD, mainly for medication that is commonly used for COPD exacerbations (i.a. prednisone). The findings of our study indicate that obese patients with milder stages of COPD are suffering more from breathing problems and exacerbations as compared to normal weight patients. As already discussed by Cecere et al.,^8^ breathing problems among these patients are possibly more related to obesity than to COPD-related factors, making medication for treatment of COPD less effective. Instead, focus on lifestyle for weight reduction would probably be more appropriate for these patients.

Previous studies mostly used a BMI of 18.5 kg/m^2^ as lowest value for the normal weight category,^3,4,8–10^ whereas in our study a BMI of 21 kg/m^2^ was set as lowest value, based on the Dutch general practitioner (GP)-guidelines for management of COPD.^18^ However, additional analyses, using BMI 18,5 kg/m^2^ as lowest value for the normal weight category, yielded similar results (data not shown).

### Strengths and limitations

While previous studies were mainly based on self-reported data, a major strength of this study is the use of routinely recorded data from general practices. In the Netherlands, the GP is mostly the first professional to consult for health problems. Therefore, the GP has a complete overview of all health problems of his/her patient population. This allowed us to examine the prevalence of all comorbid disorders of interest based on diagnoses recorded by GPs.

A consequence of using data from routine clinical practice is the limitation in data availability. For example, BMI and spirometry data were only available for respectively 44 and 17% of patients with prevalent (i.e. already diagnosed) COPD. This can partly be due to the fact that patients with severe COPD are mostly treated in secondary care by a pulmonologist. Monitoring data (e.g. on BMI and spirometry) that are performed in secondary care are probably not always registered in patient files of GPs. Nevertheless, we would have expected higher data availability, at least for BMI, since the large majority of the COPD population (~70%) is presented with mild to moderate airflow limitation,^19^ for whom the GP is mostly the primary healthcare professional in disease management. For these patients monitoring of the disease is recommended at least once a year, including the evaluation of BMI and/or weight.

The low availability of data on spirometry measurement is more plausible, since spirometry measurement for patients with prevalent mild to moderate COPD is only recommended to be performed at least once in 3 years.^18^ Moreover, for patients with mild COPD, measurement of spirometry is only recommended for patients who experience health problems or who are still smoking. Limitations in data availability on spirometry could have resulted in a selection bias. The patients with mild COPD in this study are possibly not truly representative to the mild COPD-population in real clinical practice. However, we do not think that this potential selection bias might have influenced the conclusions, since the focus of this study was on differences between weight-groups.

### Implications for further research, policy, and practice

The findings of this study underline the need to increase awareness in GPs on weight management for patients with milder stages of COPD. Moreover, the current study highlights that BMI is frequently not recorded in EHRs of patients with COPD, suggesting that weight management for these patients in general practice does not have a high priority yet.

For further research it would be interesting to investigate whether weight reduction in obese patients with milder stages of COPD is effective for increasing quality of life, and reducing the number of prescriptions for COPD-medication. In patients with asthma, weight reduction has been linked to positive outcomes, such as improvements on dyspnea, and exercise tolerance.^20^ Since symptoms of asthma are very similar to those of COPD, the promising results of weight reduction in patients with asthma support the need for research on the impact of weight loss in patients with COPD.

## Methods

### Study design

In this cross-sectional study, data were used from electronic health records of Dutch general practices that participated in the NIVEL-PCD in 2014. These practices were representative for all Dutch general practices regarding gender and age of the patient population.^21^ Electronic health records are used to record patient information on consultations, anthropometric and metabolic measurements, morbidity according to the International Classification of Primary Care - version 1 (ICPC-1), and drugs prescriptions according to the Anatomical Therapeutic Chemical (ATC) classification system.

### Population

Figure 1 shows the flow diagram of the patient selection. Initially, from 380 general practices of the NIVEL-PCD, all COPD patients were selected according to the following criteria: (1) having a recorded diagnosis of COPD (ICPC R91 and/or R95), prior to 1st January 2014 and (2) registered in the same general practice from 1st January to 31st December 2014, (3) at least one recording of BMI in 2014 and (4) at least one spirometry result in 2014, based on post-bronchodilator measurements. Patients who had a forced expiratory volume in 1 s (FEV_1_) divided by the forced vital capacity below 70% were classified as having spirometry confirmed COPD. Next, the FEV_1_ % predicted was employed to classify COPD. Mild COPD was defined as FEV_1_ ≥ 80 % predicted, and moderate COPD as FEV_1_ ≥ 50 and <80 % predicted, according to the GOLD classification.^11^ In case of multiple recordings of spirometry measurements, the highest value was selected.^22^

Per patient the mean BMI value was calculated over all available recorded BMI (or length and weight) measures in 2014. According to the mean BMI, patients with underweight were excluded. A BMI of <21 kg/m^2^ was used as a cut-off value for underweight, as this is reported as an indication for malnutrition in Dutch GP-guidelines for management of COPD.^18^ The remaining patients were categorized into the following weight-groups: normal weight (BMI ≥ 21 and <25 kg/m^2^), overweight (BMI ≥ 25 and <30 kg/m^2^), and obesity (BMI ≥ 30 kg/m^2^).

In addition, information on gender, age, smoking status, morbidity, and medication were extracted from the electronic health records for all selected patients.

### Outcome measures

#### Comorbid disorders

We established common (clusters of) comorbid disorders that are known to be associated with COPD and/or obesity according to the Dutch GP guidelines for management of COPD and management of obesity,^18,23^ including coronary heart diseases (ICPC K74-K76), stroke (ICPC K89-K90), hypertension (ICPC K86-K87), heart failure (ICPC K77); osteoarthritis (ICPC L89-L91); osteoporosis (ICPC L95); sleep disturbance (ICPC P06); anxiety disorders (ICPC P74); depression (ICPC P76); pneumonia (ICPC R81); lung carcinoma (ICPC R84), and diabetes (ICPC T90).

#### Medication

Eight classes of medication were established that were most commonly used to treat COPD, according to the GOLD recommendations and Dutch GP-guidelines for management of COPD,^11,18^ including SAMA, LAMA, SABA, LABA, ICS, medication with a combination of LABA and ICS, prednisone and antibiotics. The ATC-codes of medication belonging to the eight medication-classes are presented in Supplementary Table 2. For each medication-class a patient was classified as user if at least one prescription for a medication was recorded.

### Statistical analyses

Statistical analyses were performed using STATA 14.0. Descriptive statistics were used to present baseline characteristics. Logistic regression models were applied to evaluate the prevalence rates of comorbid disorders and prescribed medication, for the groups of overweight and obese patients, compared with the group of normal weight patients. To assess the effect of potential confounding factors, logistic multilevel analyses were performed, including a random intercept to account for clustered data of patients within general practices, and with adjustment for gender, age (years), smoking status (never/former/current), and lung function (FEV_1_ % predicted). Further analyses were conducted to examine interaction of BMI-category with smoking status (no current smoker/ current smoker) and COPD-status (mild/moderate). All tests were two-sided and the significance level was set at *p* < 0.05 and *p* < 0.10 for the main analyses and the interaction analyses, respectively.

### Ethical approval

Dutch law allows the use of electronic health records for research purposes under certain conditions. According to this legislation, neither obtaining informed consent from patients nor approval by a medical ethics committee is obligatory for this type of observational studies containing no directly identifiable data (Dutch Civil Law, Article 7:458).

### Data availability

The data sets generated and analyzed during this study are available on request by sending an e-mail to zorgregistraties@nivel.nl.

## Electronic supplementary material

Supplementary Table 1

Supplementary Table 2
