# Supplementary material for: Overweight in patients with chronic obstructive pulmonary disease needs more attention: a cross-sectional study in general practice
Source: NPJ Prim Care Respir Med. 2017 Nov 22;27:63. doi: 10.1038/s41533-017-0065-3 (PMC5700136; doi:10.1038/s41533-017-0065-3)
Supplement: Supplementary file 1 — Supplementary Table 1 [file 41533_2017_65_MOESM1_ESM.docx]

**Supplementary table 1. Odds ratios for the association of weight and comorbid disorders in patients with mild to moderate Chronic Obstructive Pulmonary Disease**

|  |  | **Normal weight**  **N=1,534** | **Overweight**  **N=2,212** | **Obesity**  **N=1,192** |
| --- | --- | --- | --- | --- |
| Diabetes | No. cases | 173 | 398 | 372 |
|  | Model 1 | ref. | 1.73 (1.42-2.09) | 3.57 (2.92-4.36) |
|  | Model 2 | ref. | 1.71 (1.39-2.10) | 3.79 (3.04-4.71) |
| Hypertension | No. cases | 558 | 976 | 670 |
|  | Model 1 | ref. | 1.38 (1.21-1.58) | 2.25 (1.92-2.62) |
|  | Model 2 | ref. | 1.37 (1.18-1.59) | 2.46 (2.07-2.93) |
| Osteoarthritis | No. cases | 225 | 429 | 318 |
|  | Model 1 | ref. | 1.40 (1.17-1.67) | 2.12 (1.75-2.56) |
|  | Model 2 | ref. | 1.45 (1.20-1.77) | 2.38 (1.92-2.95) |
| Heart failure | No. cases | 57 | 101 | 81 |
|  | Model 1 | ref. | 1.24 (0.89-1.73) | 1.89 (1.33-2.67) |
|  | Model 2 | ref. | 1.30 (0.89-1.89) | 2.32 (1.55-3.46) |
| Sleep disturbance | No. cases | 79 | 128 | 62 |
|  | Model 1 | ref. | 1.13 (0.85-1.51) | 1.01 (0.72-1.42) |
|  | Model 2 | ref. | 1.25 (0.92-1.70) | 1.16 (0.81-1.66) |
| Coronary heart disease | No. cases | 60 | 116 | 56 |
|  | Model 1 | ref. | 1.36 (0.99-1.87) | 1.21 (0.83-1.76) |
|  | Model 2 | ref. | 1.20 (0.85-1.69) | 1.15 (0.77-1.72) |
| Stroke | No. cases | 107 | 191 | 88 |
|  | Model 1 | ref. | 1.26 (0.99-1.61) | 1.06 (0.79-1.42) |
|  | Model 2 | ref. | 1.21 (0.92-1.58) | 1.13 (0.82-1.55) |
| Depression | No. cases | 98 | 112 | 65 |
|  | Model 1 | ref. | 0.78 (0.59-1.03) | 0.85 (0.61-1.17) |
|  | Model 2 | ref. | 0.94 (0.70-1.26) | 1.01 (0.72-1.42) |
| Pneumonia | No. cases | 80 | 94 | 54 |
|  | Model 1 | ref. | 0.81 (0.59-1.09) | 0.86 (0.61-1.23) |
|  | Model 2 | ref. | 0.72 (0.52-1.00) | 0.84 (0.58-1.22) |
| Anxiety disorder | No. cases | 55 | 56 | 19 |
|  | Model 1 | ref. | 0.70 (0.48-1.02) | 0.44 (0.26-0.74) |
|  | Model 2 | ref. | 0.80 (0.53-1.20) | 0.49 (0.28-0.86) |
| Osteoporosis | No. cases | 172 | 170 | 74 |
|  | Model 1 | ref. | 0.66 (0.53-0.82) | 0.52 (0.39-0.70) |
|  | Model 2 | ref. | 0.70 (0.54-0.90) | 0.51 (0.37-0.71) |
| Odds ratios are presented with their 95% confidence interval.  Model 1: crude model (N=4,938). Model 2: adjusted for clustering effect of general practice, gender, age, smoking status, and lung function (N=4,583). | | | | |
