# Supplementary material for: Overweight in patients with chronic obstructive pulmonary disease needs more attention: a cross-sectional study in general practice
Source: NPJ Prim Care Respir Med. 2017 Nov 22;27:63. doi: 10.1038/s41533-017-0065-3 (PMC5700136; doi:10.1038/s41533-017-0065-3)
Supplement: Supplementary file 2 — Supplementary Table 2 [file 41533_2017_65_MOESM2_ESM.docx]

**Supplementary table 2. Classification of medication for obstructive airway disease**

| **Medication class** | **ATC-codes** |
| --- | --- |
| SAMA | R03BB01 |
| SABA | R03AC02 |
| LAMA | R03BB04 |
| LABA | R03AC13; R03AC12; R03AC18 |
| ICS | R03BA05; R03BA08; R03BA02; R03BA01 |
| LABA + ICS | R03AK06; R03AK07; R03AK08 |
| Prednisone | H02AB06 |
| Antibiotics | J01CA04; J01AA02 |
| SAMA: short-acting muscarinic antagonist; SABA: short acting beta2-antagonist; LAMA: long-acting muscarinic antagonist; LABA: long-acting beta2-antagonist; ICS: inhaled corticosteroids | |
